# Cerebral white matter lesions and regional blood flow are associated with reduced cognitive function in early-stage cognitive impairment

**DOI:** 10.3389/fnagi.2023.1126618

**Published:** 2023-02-16

**Authors:** Takeshi Kuroda, Kenjiro Ono, Motoyasu Honma, Miki Asano, Yukiko Mori, Akinori Futamura, Satoshi Yano, Mizuki Kanemoto, Sotaro Hieda, Yasuhiko Baba, Masahiko Izumizaki, Hidetomo Murakami

**Affiliations:** ^1^Department of Neurology, Showa University School of Medicine, Tokyo, Japan; ^2^Department of Neurology, Kanazawa University Graduate School of Medical Sciences, Kanazawa, Japan; ^3^Department of Physiology, Showa University School of Medicine, Tokyo, Japan; ^4^Department of Neurology, Showa University Fujigaoka Hospital, Kanagawa, Japan

**Keywords:** cerebral white matter lesions, cerebral blood flow, cognitive impairment, path analysis, single-photon emission computed tomography, early-stage cognitive impairment

## Abstract

**Background:**

Differences in the extent of cerebral white matter lesions (WML) and regional cerebral blood flow (rCBF) in early-stage cognitive impairment (ESCI) contribute to the prognosis of cognitive decline; however, it is unclear precisely how WML and rCBF affect cognitive decline in ESCI.

**Objective:**

We examined the association between WML, rCBF, and cognitive impairment in the ESCI, using path analysis to clarify how these variables affect each other.

**Methods:**

Eighty-three patients who consulted our memory clinic regarding memory loss were included in this study based on the Clinical Dementia Rating. Participants underwent the Mini-Mental State Examination (MMSE), brain magnetic resonance imaging (MRI) for voxel-based morphometry analysis, and brain perfusion single-photon emission computed tomography (SPECT) for rCBF evaluation in cortical regions, using 3D stereotactic surface projection (3D-SSP) analysis.

**Results:**

Path analysis was performed on the MRI voxel-based morphometry and SPECT 3D-SSP data, showing a significant correlation between both and MMSE scores. In the most suitable model (GFI = 0.957), correlations were observed between lateral ventricular (LV-V) and periventricular WML (PvWML-V) volumes [standardized coefficient (SC) = 0.326, *p* = 0.005], LV-V and rCBF of the anterior cingulate gyrus (ACG-rCBF; SC = 0.395, *p* < 0.0001), and ACG-rCBF and PvWML-V (SC = 0.231, *p* = 0.041). Furthermore, a direct relationship between PvWML-V and MMSE scores was identified (SC = −0.238, *p* = 0.026).

**Conclusion:**

Significant interrelationships were observed among the LV-V, PvWML-V, and ACG-rCBF that directly affected the MMSE score in the ESCI. The mechanisms behind these interactions and the impact of PvWML-V on cognitive function require further investigation.

## 1. Introduction

The interaction between cerebral white matter lesions (WML), regional cerebral blood flow (rCBF), and cognitive impairment in dementia has recently begun receiving attention. WML are common in patients with dementia and elderly individuals with normal cognition. These lesions are identified as white matter (WM) hyperintensities on T2-weighted fluid-attenuated inversion recovery (FLAIR) magnetic resonance imaging (MRI) scans (Weller et al., 2015). WML are divided into periventricular (PvWML) and deep WM lesions (DWML; Kim et al., 2008), which have different pathogenic mechanisms. According to postmortem studies, PvWML show discontinuous ependyma with high extracellular fluid content, gliosis, and myelin loss; conversely, DWML exhibit greater axonal loss, vacuolation, and increased tissue loss in more severe lesions, suggesting infarction in addition to demyelination and gliosis (Wardlaw et al., 2015; Arvanitakis et al., 2017). In previous studies, WML was associated with loss of cognitive function and increased risk of dementia (Wang et al., 2020). The influence of WML on the development of dementia is hypothesized to differ according to brain region, a theory that has recently received much research attention. PvWML may have a larger effect than DWML on all-cause dementia; however, findings on the effects of WML on cognitive impairment and dementia are inconsistent (Kim et al., 2015). Additionally, conversion from normal cognition or mild cognitive impairment (MCI) to Alzheimer’s disease (AD) is hypothesized to be associated with the severity of PvWML (Lee et al., 2014; Kim et al., 2015).

The assessment of rCBF is important in diagnosing and understanding the pathophysiology of dementia. In clinical practice, brain perfusion single-photon emission computed tomography (SPECT) and positron emission tomography (PET) are functional neuroimaging techniques used to evaluate the decrease in cortical perfusion in the brain (Pimlott and Ebmeier, 2007). Of these, SPECT is more often used in Japan to diagnose dementia clinically. SPECT and PET could be useful tools for diagnosing different types of dementia, such as AD, vascular dementia (VaD), dementia with Lewy bodies (DLB), frontotemporal dementia (FTD), and idiopathic normal pressure hydrocephalus (iNPH), by observing different CBF patterns. Recently, the evaluation of rCBF using MRI arterial spin labeling has also proven helpful in observing decreased rCBF patterns in patients with dementia with minimum invasion (Hays et al., 2016). Several investigations have demonstrated rCBF abnormalities that predicted the early stages of MCI and AD using SPECT and PET (Minoshima et al., 1997; Mosconi et al., 2005; Newberg and Alavi, 2005; Nobili et al., 2009; Habert et al., 2011). Specifically, studies using ^123^iodoamphetamine (IMP)-SPECT reported that hypoperfusion in the posterior cingulate cortex, precuneus, and temporoparietal lobe in patients with MCI was predictive of conversion to AD (Ito et al., 2013). Therefore, specific rCBF abnormalities identified using SPECT may be useful predictors for distinguishing AD converters from non-converters. However, since cerebral hypoperfusion in the preclinical stage of AD or in MCI has been reported in many regions, including the frontal, temporal, and parietal lobes and the anterior cingulate gyrus, it remains unclear which areas of hypoperfusion are the most important indicators of dementia progression (Hays et al., 2016).

White matter lesion and rCBF are closely related. Physiological changes within the visible WML and the surrounding healthy-looking WM, or the WML penumbra, have garnered increasing interest. Similar to structural WML penumbras, rCBF penumbras have shown an association with the expansion of WML over time (Promjunyakul et al., 2016). Further, when comparing PvWML and DWML, PvWML growth was more closely associated with rCBF than that of DWML (Promjunyakul et al., 2018). Therefore, WML, rCBF, and cognitive decline are considered closely related in dementia, especially in the early stages of illness. Studies have shown that AD and MCI with the presence of WML are accompanied by severe and widespread rCBF reduction related to more rapid worsening of Mini-Mental State Examination (MMSE) scores (Hanaoka et al., 2016; Ishibashi et al., 2018).

White matter lesion and rCBF in early-stage cognitive impairment (ESCI), such as subjective cognitive impairment (SCI), MCI, and mild dementia, may play a role in the prognosis of cognitive decline; however, the extent of this effect remains unclear. If noninvasive imaging tests such as MRI and SPECT could be used to determine the degree of future cognitive decline in ESCI, this would enable early detection of dementia; additionally, these tools could aid in selecting patients requiring invasive testing, such as lumbar puncture for collection of cerebrospinal fluid (CSF) biomarkers. However, no previous studies have examined the causal effects of WML and rCBF on cognitive decline in ESCI using background data from MRI and SPECT to understand how general imaging techniques can reveal the neurobiology of cognitive decline. In this study, we examined the association between WML (measured using MRI), rCBF (assessed using SPECT), and cognitive impairment, using path analysis to clarify the extent to which WML and/or CBF may affect cognitive function in the early stages of all-cause cognitive impairment.

## 2. Materials and methods

### 2.1. Patients

**Figure 1:**
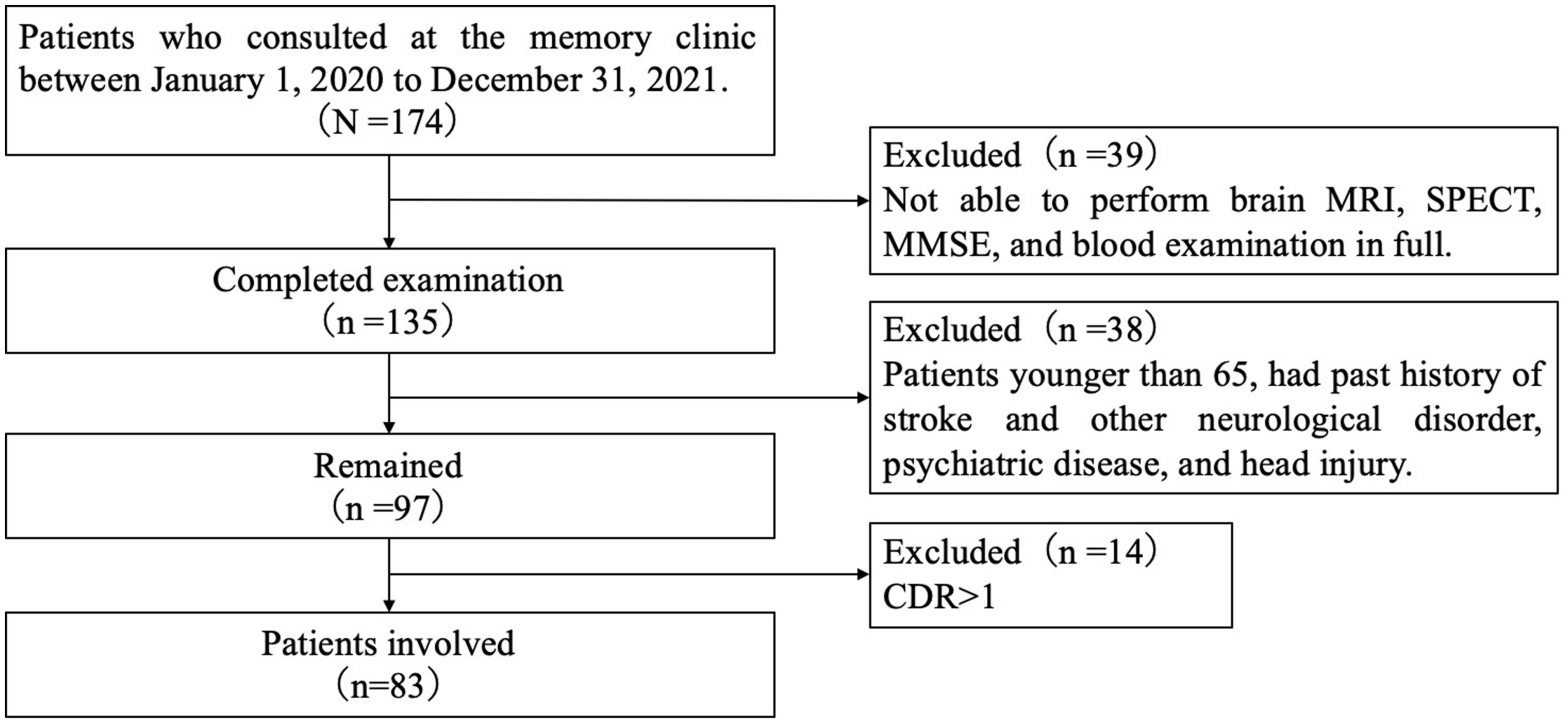
Flowchart of patient recruitment. MRI, magnetic resonance imaging; SPECT, single-photon emission computed tomography; MMSE, mini-mental state examination japanese version; and CDR, clinical dementia rating.

We included patients who consulted the memory clinic of the Department of Neurology at the Showa University School of Medicine in Japan between 1 January 2020 and 31 December 2021, for memory loss. Of those who were able to undergo brain MRI, rCBF SPECT, the Japanese version of the MMSE, blood testing, and apolipoprotein E (*APOE*) genotyping in full, we excluded patients aged <65 years and those with a history of symptomatic stroke and other neurological disorders, with a history of treatment for psychiatric illness, or head injuries. Since this study aimed to examine the association between WML and rCBF in the early stages of cognitive impairment, we wanted to exclude patients with increased WML volume or reduced rCBF as a result of symptomatic stroke. Here, we excluded patients with a history of psychiatric illness to eliminate the potential confound of pseudodementia, which is characterized by cognitive impairment secondary to depression, delusional disorder, or the use of psychiatric medications. This is important as we aim to specifically study the behavioral and psychological symptoms of dementia, such as depression, apathy, and visual hallucinations, in patients whose cognitive impairment may progress in the future. Finally, a total of 83 patients with a global clinical dementia rating (CDR) score ≤ 1 were included in the study as the ESCI group (Figure 1). All the patients underwent a clinical interview and neurological examination by a trained neuropsychologist. Additionally, we included 10 participants without cognitive impairment or memory complaints who were able to undergo all examinations as healthy controls (HC).

Although the global CDR score has been used in screening SCI, MCI, and mild dementia in many clinical trials or studies, its reliability is suboptimal in very mild dementia cases with CDR = 0.5. However, many studies have indicated that the sum of boxes score of the CDR (CDR-SB) can provide additional information to the CDR in detecting very mild dementia and distinguishing these patients from those with MCI. Additionally, we calculated CDR-SB and classified it as 0 = normal, 0.5–2.5 = questionable impairment, 3.0–4.0 = very mild dementia, and 4.5–9.0 = mild dementia (O'Bryant et al., 2008; Tzeng et al., 2022). SCI was characterized by self-reported memory complaints, CDR score of 0 or 0.5 and CDR-SB score of 0–2.5, MMSE scores within the normal range for cognition, and no evidence of impairment in functional activities. MCI diagnosis was based on the MCI diagnostic criteria proposed by Petersen (2004): memory complaint corroborated by an informant, global CDR score of 0.5 and CDR-SB score of 0.5–2.5, a cognitive decline in MMSE, but no evidence of impairment in functional activities revealed by clinical interview. Diagnoses of AD, VaD, DLB, and iNPH were made according to the guidelines of the National Institute on Aging-Alzheimer’s Association workgroups (McKhann et al., 2011), the diagnostic criteria for vascular cognitive disorders of the International Society for Vascular Behavioral and Cognitive Disorders (Sachdev et al., 2014), the revised criteria for the clinical diagnosis of DLB (McKeith et al., 2017), and the third edition of the Japanese Guidelines for Management of iNPH (Nakajima et al., 2021), respectively. This study was approved by the Ethics Committee of Showa University School of Medicine and conducted in accordance with the principles of the Declaration of Helsinki (as revised in 2013). All participants provided written informed consent.

### 2.2. Magnetic resonance imaging and voxel-based morphometry analysis

Structural MRI scans were conducted using a 1.5 T MR scanner (Magnetom Essenza; Siemens, Munich, Germany). The high-resolution T1-weighted 3D images of the whole brain (144 sagittal slices; 1.0 × 1.0 × 1.25 mm^3^; repetition time = 1,600 ms; echo time = 4.7 ms; flip angle = 15°; and field of view = 256 × 256) and transverse FLAIR sequences with 6 mm slice thickness were acquired for each patient. We used Brain Anatomical Analysis using Diffeomorphic deformation (BAAD) version 4.2 software to evaluate the extent and distribution of WML. BAAD is voxel-based morphometry (VBM)-supported software based on Statistical Parametric Mapping 12 (Syaifullah et al., 2021). The brain was extracted from the 3D MR images by skull stripping, segmented into gray matter (GM), WM, and CSF, and warped into the Montreal Neurological Institute space using the Diffeomorphic Anatomical Registration through Exponentiated Lie Algebra algorithm. Voxel volume was converted into a voxel signal by a “modulation” procedure. Finally, the data were smoothed to obtain smoothed, modulated, warped, and segmented images. The BAAD version used in this study interpolates FLAIR images with 3D T1-weighted images to automatically correct WML contamination in the GM and calculates the volume of the WML and lateral ventricles. Moreover, it can extract and measure the volume of the WML in the deep WM, periventricular, and corpus callosum areas. Further details of the VBM processing flow in the BAAD software are provided elsewhere (Syaifullah et al., 2021). The following volumes were assessed: total intracranial, GM (GM-V), WM (WM-V), lateral ventricular (LV-V), CSF (CSF-V), total WML (TWML-V), PvWML (PvWML-V), DWML (DWML-V), frontal lobe WML (fWML-V), temporal lobe WML, parietal lobe WML, and occipital lobe WML volumes.

### 2.3. SPECT and 3D stereotactic surface projection analysis

Brain perfusion SPECT data were obtained using three head gamma cameras (GCA-9300R; Canon Medical Systems, Tochigi, Japan) with an ultra-high-resolution fan-beam collimator. SPECT images were acquired 15 min after the injection of a 222 MBq dose of ^123^I-IMP (Nihon Medi-Physics Co. Ltd., Tokyo, Japan). Projection data were obtained for 20 min. Imaging parameters were as follows: matrix size = 128 × 128; pixel size = 2.9 mm; slice thickness = 2.9 mm; energy window = 159 keV ± 20%. Data were reconstructed using the filtered back-projection method with a Butterworth filter (0.1 cycle/pixel). SPECT images obtained were analyzed using 3D stereotactic surface projection (3D-SSP) to generate Z-score maps in the iSSP software included in “medi+FALCON version 1.2” (Nihon Medi-Physics Co. Ltd., Tokyo, Japan). After anatomical standardization, the cortical count information was projected onto the brain surface to create a brain surface blood flow image, which was compared with those in a database created from the data of multiple healthy individuals. The *Z*-score was used to display images of areas that deviated from the healthy average, and the percentage of hypoperfusion pixels with *Z*-scores >2 were calculated for the following cortical regions: anterior, middle, medial, and inferior frontal gyri; precentral gyrus; paracentral lobule; orbital gyrus; straight gyrus; area subcallosal; postcentral gyrus; superior and inferior parietal lobules; angular gyrus; supramarginal gyrus; superior, middle, and inferior temporal gyri; fusiform gyrus; parahippocampal gyrus; uncinate gyrus; superior, middle, and inferior occipital gyri; anterior and posterior cingulate gyri; cingulate gyrus; precuneus; cuneus; lingual gyrus; and thalamus. The details of 3D-SSP have been summarized elsewhere (Minoshima et al., 1995).

### 2.4. General risk factors for white matter lesions and dementia

Laboratory tests were performed to evaluate risk factors for WML. Blood samples were assessed for lipid metabolism (total cholesterol, low- and high-density lipoprotein cholesterol, and triglycerides) and glucose metabolism (hemoglobin A1c); further, DNA was extracted for *APOE* genotyping. We applied the Invader® assay for screening two mutations (C112R: TGC → CGC, R158C: CGC → TGC) of the *APOE* gene by BML Laboratories, Saitama, Japan. The Invader assay combines structure-specific cleavage enzymes and a universal fluorescent resonance energy transfer system. Six patterns of *APOE* genotypes (ε2/ε2, ε2/ε3, ε2/ε4, ε3/ε3, ε3/ε4, and ε4/ε4) were determined by combining two mutations. Hypertension, hyperlipidemia, diabetes, current smoking status, and alcohol consumption were recorded from self-reported medical history and/or medication use data.

### 2.5. Statistical analyses

An unpaired *t*-test and Chi-square test were performed to examine the group differences between ESCI and HC individuals. Pearson’s correlation analysis was used to identify associations between the MMSE scores and the MRI VBM and SPECT 3D-SSP analysis data. Additionally, path analysis was performed to test the goodness-of-fit index (GFI). The VBM and SPECT data were corrected for age and sex. Statistical significance was defined as *p* < 0.05. SPSS 26.0 (IBM Corp., Armonk, NY, United States) was used for the unpaired t-test, Chi-square test, and Pearson’s correlation analysis. SPSS Amos 27.0 (IBM Corp., Armonk, NY, United States) was used for the pass analysis.

## 3. Results

### 3.1. Demographic and clinical data

The mean age of the 83 ESCI patients was 80.3 years, and 51 (61.4%) were female (Table 1); mean global CDR and CDR-SB scores were significantly higher (0.7 vs. 0 and 2.7 vs. 0), and MMSE scores were significantly lower (23.1 vs. 28.5) than in HC. The range of CDR-SB for total patients was 0.5–8.0; 0.5–2.5 (questionable impairment), 3.0–4.0 (very mild dementia), and 4.5–9.0 (mild dementia) for 47, 15, and 21 patients, respectively. Regarding the clinical diagnosis, 36, 33, 4, 4, 4, and 2 patients had MCI (43%), AD (40%), SCI (5%), VaD (5%), iNPH (5%), and DLB (2%), respectively (Table 2). *APOE* ε4 retention was significantly higher in the ESCI group than in the HC group (54.2 vs. 10.0%; Table 1). There was no significant difference in lipid metabolism (total cholesterol, low- and high-density lipoprotein cholesterol, and triglycerides) and glucose metabolism (hemoglobin A1c), as well as the percentages of hypertension, dyslipidemia, diabetes, smoking, and alcohol consumption between ESCI and HC.

**Table 1:** General white matter lesion and dementia risk factors and MRI and SPECT analysis data in early-stage cognitive impairment.

Groups (*n*)	HC (10)	S.D.	ESCI (83)	S.D.	*p*-value
Age	76.6	6.5	80.3	6.1	n.s.
Female (%)	70.0		61.4		n.s.
Global CDR	0	0	0.7	0.3	<0.0001
CDR-SB	0	0	2.7	2.1	<0.0001
MMSE	28.5	0.8	23.1	4.3	<0.0001
**General risk factors for WML and dementia**					
ApoE ε4 (%)	10.0		54.2		0.03
T-Chol (mg/dl)	203.4	34.8	213.8	34.8	n.s.
TG (mg/dl)	134.7	49.3	140.4	98.2	n.s.
LDL-C (mg/dl)	116.9	27.7	120.6	28.0	n.s.
HDL-C (mg/dl)	59.6	10.6	65.2	17.7	n.s.
HbA1c (%)	6.25	1.1	6.2	1.0	n.s.
Hypertension (%)	50.0		50.6		n.s.
Dyslipidemia (%)	30.0		59.0		n.s.
Diabetes (%)	10.0		24.1		n.s.
Smoking (%)	10.0		18.1		n.s.
Alcohol (%)	10.0		38.6		n.s.
**MRI VBM analysis data (mL)**					
LV-V	38.8	9.5	53.1	21.5	0.002
GM-V	528.3	24.8	507.7	31.5	0.04
WM-V	465.8	26.7	446.7	34.3	0.07
CSF-V	468.3	40.6	509.0	51.6	0.02
TWML-V	8.0	7.3	19.7	16.9	<0.001
DWML-V	2.8	3.2	9.5	11.4	<0.001
PvWML-V	5.2	4.3	10.3	6.3	0.007
fWML-V	1.1	1.2	2.2	3.0	0.04
tWML-V	0.2	0.2	0.3	0.4	n.s.
pWML-V	1.0	1.3	1.7	2.0	n.s.
oWML-V	0.4	0.5	0.4	0.4	n.s.
**SPECT 3D-SSP analysis data Mean percentage (%) of decreased rCBF (*Z*-score > 2) pixel**	
Anterior frontal gyrus	13.4	15.2	20.2	21.1	n.s.
Middle frontal gyrus	14.8	11.8	23.5	20.7	n.s.
Inferior frontal gyrus	17.5	14.0	32.4	28.1	0.02
Precentral gyrus	4.3	4.4	8.7	11.7	0.03
Medial frontal gyrus	13.4	18.4	21.9	22.6	n.s.
Paracentral lobule	3.0	5.8	4.9	12.5	n.s.
Orbital gyrus	3.6	10.9	15.6	31.2	0.02
Straight gyrus	1.9	5.7	6.0	16.9	n.s.
Area subcallosal	5.0	15.0	15.7	24.7	n.s.
Postcentral gyrus	10.4	12.3	12.3	15.0	n.s.
Superior parietal lobule	14.3	14.7	20.0	21.1	n.s.
Inferior parietal lobule	9.9	7.0	26.7	29.2	< 0.0001
Angular gyrus	12.9	13.1	53.8	68.0	< 0.0001
Supramarginal gyrus	8.4	11.8	39.1	47.5	< 0.0001
Superior temporal gyrus	26.2	21.3	43.3	34.1	n.s.
Middle temporal gyrus	12.3	11.8	30.4	30.9	0.002
Inferior temporal gyrus	9.8	10.0	21.7	29.2	0.02
Fusiform gyrus	9.1	11.3	12.1	18.2	n.s.
Parahippocampal gyrus	14.9	23.5	14.3	20.4	n.s.
Uncinate gyrus	13.9	14.0	29.6	38.2	0.02
Superior occipital gyrus	10.0	15.6	12.9	25.8	n.s.
Middle occipital gyrus	2.6	3.4	7.4	20.3	n.s.
Inferior occipital gyrus	5.5	9.2	6.7	20.7	n.s.
Anterior cingulate gyrus	19.2	18.0	58.3	41.9	< 0.0001
Cingulate gyrus	12.5	12.0	35.7	29.3	0.00015
Posterior cingulate gyrus	8.5	9.0	25.4	35.3	0.0012
Precuneus	18.4	22.1	33.4	34.5	n.s.
Cuneus	6.7	8.8	9.9	16.1	n.s.
Lingual gyrus	2.1	4.2	6.2	22.2	n.s.
Thalamus	0.0	0.0	4.2	12.7	0.004

ESCI, early-stage cognitive impairment; HC, healthy controls; SD; standard deviation; n.s., not significant; CDR-SB, Clinical Dementia Rating Sum of Boxes; MMSE, Mini-Mental State Examination, Japanese version; WML, white matter lesions; APOE, apolipoprotein E gene; T-Chol, total cholesterol; TG, triglycerides; LDL-C, low-density lipoprotein cholesterol; HDL-C, high-density lipoprotein cholesterol; HbA1c, hemoglobin A1c; MRI, magnetic resonance imaging; VBM, voxel-based morphometry; LV-V, lateral ventricular volume; GM-V, gray matter volume; WM-V, white matter volume; CSF-V, cerebrospinal fluid volume; TWML-V, total white matter lesion volume; DWML-V, deep white matter lesion volume; PvWML-V, periventricular white matter lesion volume; fWML-V, frontal lobe white matter lesion volume; tWML-V, temporal lobe white matter lesion volume; pWML-V, parietal lobe white matter lesion volume; oWML-V, occipital lobe white matter lesion volume; SPECT, single-photon emission computed tomography; 3D-SSP, 3D stereotactic surface projection; and rCBF, regional cerebral blood flow.

**Table 2:** Clinical diagnosis of patients with early-stage cognitive impairment.

Clinical diagnosis (n)	HC (10)	S.D.	SCI (4)	S.D.	MCI (36)	S.D.	AD (33)	S.D.	VaD (4)	S.D.	iNPH (4)	S.D.	DLB (2)	S.D.
Age	76.6	6.5	71.8	8.4	81.4	4.9	79.8	6.2	85.0	3.1	83.0	3.1	72.5	4.5
Female (%)	70.0		100.0		64.0		58.0		50.0		25.0		100.0	
Global CDR	0	0	0.3	0.3	0.5	0.0	0.8	0.2	0.8	0.3	0.8	0.3	1.0	0.0
CDR-SB	0	0	0.5	0.0	1.0	0.5	4.4	1.5	3.6	0.7	3.3	2.1	6.0	1.0
MMSE	28.5	0.8	28.8	1.3	25.5	2.4	20.5	4.0	20.3	2.5	22.3	3.3	18.0	4.0

HC, healthy controls; SD; standard deviation; SCI, subjective cognitive impairment; MCI, mild cognitive impairment; AD, Alzheimer’s disease; VaD, vascular dementia; iNPH, idiopathic normal pressure hydrocephalus; DLB, dementia with Lewy bodies; CDR-SB, clinical dementia rating sum of boxes; and MMSE, mini-mental state examination, Japanese version.

### 3.2. MRI and voxel-based morphometry analysis

Lateral ventricular-volume (LV-V) and CSF-V were significantly larger, while GM-V and WM-V were significantly smaller in ESCI than in HC. The volumes of the TWML-V, DWML-V, PvWML-V, and fWML-V were significantly larger in the ESCI group than in the HC group. Regarding distribution, the frontal lobe WML volume tended to be the largest, and the PvWML-V tended to be greater than the DWML-V in both groups (Table 1).

### 3.3. SPECT and 3D stereotactic surface projection analysis

**Figure 2:**
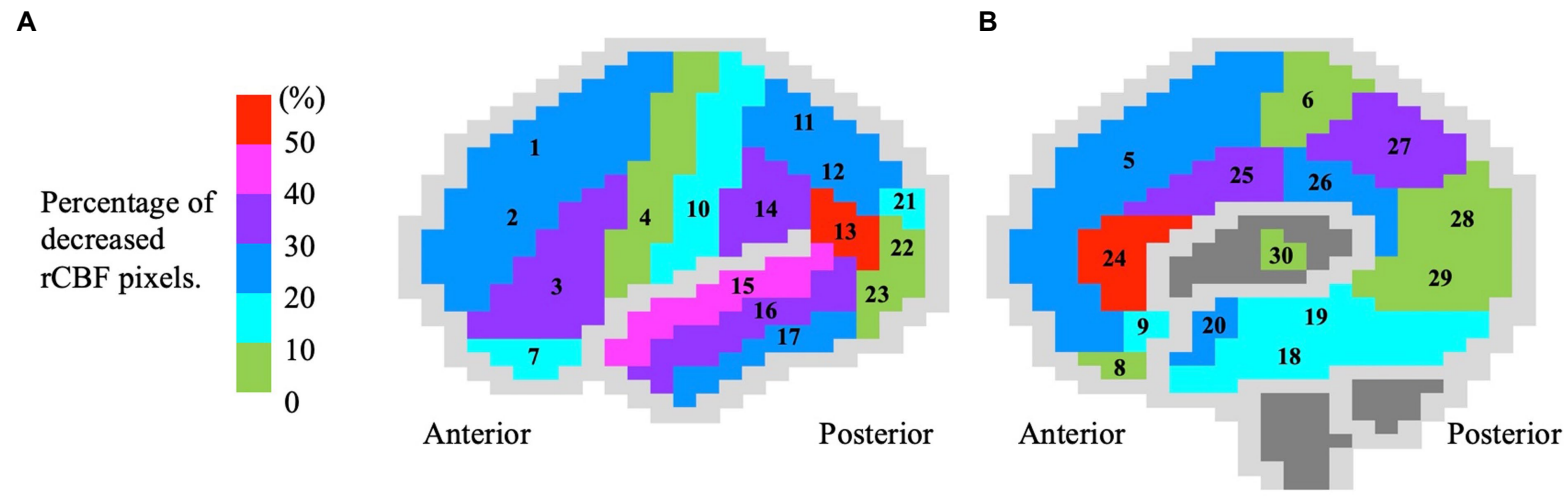
Color brain mapping showing the distribution of decreased regional cerebral blood flow in 83 early-stage cognitive impairment patients. The figure shows the **(A)** lateral and **(B)** medial sides of the brain. The brain gyrus is colored according to the mean percentage (%) of rCBF reduction (*Z*-score > 2), corresponding to the colored bar. The numbers in the figure correspond to the following brain gyrus, (1) anterior frontal gyrus; (2) middle frontal gyrus; (3) inferior frontal gyrus; (4) precentral gyrus; (5) medial frontal gyrus; (6) paracentral lobule; (7) orbital gyrus; (8) straight gyrus; (9) area subcallosal; (10) postcentral gyrus; (11) superior parietal lobule; (12) inferior parietal lobule; (13) angular gyrus; (14) supramarginal gyrus; (15) superior temporal gyrus; (16) middle temporal gyrus; (17) inferior temporal gyrus; (18) fusiform gyrus; (19) parahippocampal gyrus; (20) uncinate gyrus; (21) superior occipital gyrus; (22) middle occipital gyrus; (23) inferior occipital gyrus; (24) anterior cingulate gyrus; (25) cingulate gyrus; (26) posterior cingulate gyrus; (27) precuneus; (28) cuneus; (29) lingual gyrus; and (30) thalamus. rCBF, regional cerebral blood flow.

In the SPECT 3D-SSP analysis data, the percentage of pixels with decreased rCBF (*Z*-score > 2) was significantly higher in the ESCI patients than in the HC in the inferior frontal gyrus, precentral gyrus, orbital gyrus, inferior parietal lobule, angular gyrus, supramarginal gyrus, middle and inferior temporal gyri, uncinate gyrus, anterior and posterior cingulate gyri, cingulate gyrus, and thalamus (Table 1). The mean percentage of pixels with decreased rCBF (Z-score > 2) in the ESCI group is shown in the colored figure (Figure 2). The anterior cingulate gyrus and angular gyrus had more than 50% of pixels with decreased rCBF, followed by the superior temporal gyrus.

### 3.4. Voxel-based morphometry and 3D stereotactic surface projection analysis data correlations with MMSE in early-stage dementia

**Figure 3:**
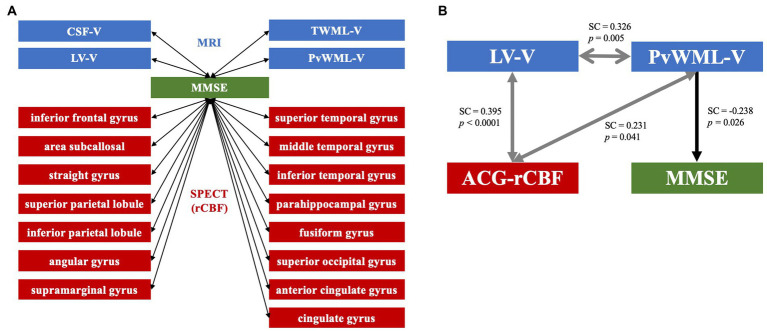
**(A)** MRI and SPECT data showing correlations with MMSE in early-stage cognitive impairment. The LV-V, CSF-V, TWML-V, and PvWML-V from MRI voxel-based morphometry analysis showed significant correlations with MMSE scores. The percentage of pixels with decreased rCBF (*Z*-score > 2) in the regions shown in the figure correlated with MMSE scores in the SPECT 3D-SSP analysis. The arrows in both directions indicate correlation. **(B)** Path diagram. Path analysis suggested that there were interrelationships between the LV-V, PvWML-V, and ACG-rCBF, and that the PvWML-V directly affected the MMSE score. Arrows in both directions indicate interrelationships between factors, while unidirectional arrows indicate influence in that direction. MRI, magnetic resonance imaging; SPECT, single-photon emission computed tomography; MMSE, Mini-Mental State Examination; CSF-V, cerebrospinal fluid volume; TWML-V, total white matter lesion volume; LV-V, lateral ventricular volume; PvWML-V, periventricular white matter lesion volume; rCBF, regional cerebral blood flow; SC, standardized coefficient; ACG-rCBF, regional cerebral blood flow in the anterior cingulate gyrus; and 3D-SSP, 3D stereotactic surface projection.

The results of the correlation analysis between MRI VBM data and cognitive measures (MMSE, LV-V, CSF-V, TWML-V, and PvWML-V) revealed a statistically significant negative association with MMSE scores (Figure 3A). The cortical regions in which the percentage of pixels with decreased rCBF (Z-score > 2), measured by SPECT 3D-SSP analysis, showed a significant correlation with MMSE were the inferior frontal gyrus; straight gyrus; subcallosal area; superior and inferior parietal lobules; angular gyrus; supramarginal gyrus; superior, middle, and inferior temporal gyri; fusiform gyrus; parahippocampal gyrus; superior occipital gyrus; anterior cingulate gyrus; and cingulate gyrus (Figure 3A).

### 3.5. Path analysis

Path analysis was performed on the MRI VBM analysis and SPECT 3D-SSP analysis data, showing a significant correlation with the MMSE scores. In the most suitable model (GFI = 0.957), correlations were identified between LV-V and PvWML-V [standardized coefficient (SC) = 0.326, *p* = 0.005], LV-V and the anterior cingulate gyrus rCBF (ACG-rCBF; SC = 0.395, *p* < 0.0001), and between ACG-rCBF and PvWML-V (SC = 0.231, *p* = 0.041). Moreover, the analysis revealed a direct relationship between PvWML-V and MMSE scores (SC = −0.238, *p* = 0.026; Figure 3B).

## 4. Discussion

The present study examined the association between WML, rCBF, and cognitive impairment using path analysis to clarify how WML and/or CBF affects cognitive function in the early stages of all-cause cognitive impairment, including SCI, MCI, and mild dementia. The path analysis findings suggest the presence of interrelationships among the LV-V, PvWML-V, and ACG-rCBF variables and that the PvWML-V directly affected the MMSE scores in this study.

The enlargement of LV-V in dementia and aging may reflect brain atrophy and CSF/interstitial fluid (ISF) circulation. iNPH is the most well-known form of dementia associated with LV-V enlargement based on CSF/ISF stasis (Adams et al., 1965; Ishikawa et al., 2016). The glymphatic system is the CSF/ISF transport system that facilitates extracellular waste removal through a network of astroglia-supported perivascular or perineural channels that drain into the cervical and basal meningeal lymphatic networks or the major dural sinuses. Recent reports suggest that this system exists in the human brain and is impaired in patients with some types of dementia, such as iNPH and AD (Iliff et al., 2012; Aspelund et al., 2015; Reeves et al., 2020). Although ESCI consists of AD, iNPH, and all-cause dementia, LV-V and CSF-V were significantly larger in ESCI patients than in HC. This result suggests that enlargement of LV-V in the early stages of all-cause cognitive impairment might result from CSF/ISF stasis. Although it has long been recognized that ventricular enlargement in dementia is associated with brain atrophy, assuming that impairment of excretory waste systems, such as the glymphatic system, is commonly involved in the background of various dementias, the enlargement of LV-V in ESCI may also be affected by CSF/ISF stasis.

In contrast, PvWML reflects vasogenic edema and gliosis caused by obstruction of the periventricular medullary veins and insufficiency of ISF circulation (Black et al., 2009). In a previous prospective cohort study, PvWML-V at baseline was associated with a greater increase in LV-V and a decrease in GM-V (Kloppenborg et al., 2012). In our previous study, we also reported a positive correlation between LV-V and PvWML-V in healthy participants and in those with AD and iNPH (Kuroda et al., 2022). Therefore, enlargement of the PvWML-V in dementia may also be an indication of CSF/ISF stasis; consequently, LV-V and PvWML-V may be related to each other *via* CSF/ISF stasis.

In this study, we found an association between LV-V, PvWML-V, and ACG-rCBF in the ESCI group. Periventricular WM rCBF is reduced in diseases associated with the enlargement of LV-V and PvWML-V. A previous study on iNPH using ^123^IMP-SPECT and MRI arterial spin labeling showed reduced perfusion around the corpus callosum, especially frontal-dominant, including the ACG (Sasaki et al., 2007; Virhammar et al., 2017). Additionally, a study examining the relationship between PvWML and rCBF showed that rCBF tends to decrease inside the WM and in the healthy-appearing WM surrounding PvWML. Another study that evaluated the association between rCBF and WML growth using MRI arterial spin labeling and diffusion tensor imaging showed that lower baseline rCBF was associated with the generation of a new PvWML (Promjunyakul et al., 2018). Thus, an increase in the LV-V and PvWML-V is thought to be closely related to a greater reduction in rCBF in the periventricular region. While the cingulate gyrus is the closest to the lateral ventricles *via* the corpus callosum, and the ACG surrounds the anterior part of the corpus callosum, ACG-rCBF is susceptible to the increase in LV-V and PvWML-V. The present study also suggested a significantly decreased rCBF-ACG in the ESCI group compared with that in HC, suggesting that anterior cingulate cortex metabolism may be important in the early stages of cognitive impairment. Recently, metabolic maintenance of the anterior cingulate cortex has been considered a crucial signature of “cognitive resilience.” Among the oldest, those who maintain normal cognition may be protected from dementia and may provide a model to identify the brain mechanisms underlying cognitive resilience. As reported in one recent study, FDG-PET uptake in the bilateral anterior cingulate cortex and the anterior temporal pole was one of the resilience indicators, indicating that these areas are fundamental to maintaining global cognition in all cognitively stable participants over 80 years old (Arenaza-Urquijo et al., 2019). Although reduced rCBF in the ACG in ESCI patients has not received much attention, it may be an important finding that suggests progressive cognitive decline.

The results of the path analysis suggested that PvWML-V directly affected the MMSE scores. Many previous studies have suggested that WML are a risk factor for cognitive impairment in dementia, mostly in AD and VaD (Hu et al., 2021). Several longitudinal studies have suggested that WML confer an increased risk of dementia, independent of hippocampal atrophy or amyloid pathology (Brickman et al., 2012; Provenzano et al., 2013). Especially in AD, there has been a focus on the association with amyloid accumulation as a cause of WML. A systematic review suggests that amyloid accumulation and WML are independent but additive processes (Roseborough et al., 2017). A recent cross-sectional study using 11C-labeled Pittsburgh Compound B-positron emission tomography (PiB-PET) has revealed that higher WML grades on MRI significantly correlate with lower accumulation of amyloid pathology among patients with AD, suggesting that additional cerebral ischemic damage in AD could accelerate cognitive decline and facilitate early recognition of dementia (Kasahara et al., 2019). In addition to AD and VaD, WML are also thought to be a risk factor for cognitive impairment in PD and DLB (Mirza et al., 2019; Butt et al., 2021). Although the impact of WML on cognitive function remains unclear, its influence on the development of dementia is thought to differ according to location. In the present study, although TWML-V, DWML-V, and PvWML-V were all significantly larger in the ESCI group than in HC, the results of path analysis showed that PvWML-V directly affected MMSE scores in ESCI patients. Similar results have been obtained in previous studies, showing that PvWML has a larger effect than DWML-V on the development of all-cause dementia (Kim et al., 2015). Pathological studies have hypothesized that DWML changes present with more hypoxic/ischemic damage, whereas PvWML may have a greater inflammatory/metabolic component (Fernando et al., 2006). While PvWML tends to increase after the age of 65 years, it is suggested that PvWML is linked to age-related neurodegenerative processes and is associated with decreased GM-V and cognitive decline (Habes et al., 2016; Griffanti et al., 2018). Secondary loss of GM, resulting in cortical thinning and neurodegeneration, is speculated to be the cause of WML’s impact on cognitive function (Wardlaw et al., 2019). Recent studies have highlighted the importance of WML location in relation to cognitive functioning, suggesting that disruption of WM tracts is the key mechanism affecting cognition (Prins and Scheltens, 2015). One study identified that the degree of WML along the anterior thalamic radiation and forceps minor are most strongly associated with cognitive impairment and that WML volumes in individual tracts explained more cognitive impairment than the total WML burden (Biesbroek et al., 2016). WM tracts running through the brain are broadly classified into commissural, association, and projection fibers; however, major WM tracts, such as anterior thalamic radiation and forceps minor, pass through the brain’s center, which is the periventricular area. From this perspective, PvWML is more likely to affect major WM tracts than DWML and could cause secondary GM atrophy leading to cognitive decline.

In summary, our findings suggest a link between WML, CBF, and cognitive impairment in ESCI. Specifically, we posit that the enlargement of LV-V and PvWML-V, as well as a decreased ACG-rCBF, may be related to CSF/ISF stasis caused by age-related decline in clearance systems, such as the glymphatic system. Furthermore, PvWML is presumed to affect cognitive impairment due to GM-V reduction by disrupting WM tracts. However, path analysis did not suggest GM-V directly affected the MMSE scores. A more thorough study protocol is needed to elucidate the direct cause of cognitive impairment, which may be difficult to explain by a specific cause like amyloid pathology accumulation in AD. One of the limitations of this study is that, although it was cross-sectional, a longitudinal study is needed to examine the relationship between WML, rCBF, and cognitive function. Another limitation is that cognitive function was assessed using only the MMSE. While the MMSE is the most well-known and simplified cognitive assessment scale used in clinical practice, it does not have high sensitivity and specificity for assessing cognitive function in ESCI. For example, the Montreal Cognitive Assessment consists of a wide range of tasks to evaluate frontal, temporal, and parietal functions and is more suitable for screening MCI. It can accurately detect MCI with a sensitivity of 80–100% and specificity of 50–87% when the cutoff is 25 points or less (Nasreddine et al., 2005). Thus, it is advisable to use a combination of tests to assess the ESCI. Additionally, more detailed psychological tests should be used to independently evaluate memory, visuospatial cognition, and frontal lobe function to clarify the relationship between PvWML-V and cognitive impairment. The present study suggests that ACG-rCBF plays a crucial role in ESCI. The cingulate cortex is one of the largest parts of the limbic lobe. The anterior part plays a role in emotion and motor functions, while the posterior region is involved in visuospatial and memory functions (Devinsky et al., 1995). Therefore, we need to assess behavioral and psychological symptoms using a more detailed testing method to clarify the importance of ACG-rCBF changes in ESCI.

In conclusion, the results of the path analysis suggested that LV-V, PvWML-V, and ACG-rCBF influence each other in the early stages of all causes of cognitive impairment, and factors such as cerebral atrophy and CSF/ISF stasis may be intertwined as a complex background pathophysiology. Additionally, the expansion of the PvWML-V was considered to have the greatest impact on the decline in MMSE scores. The pathophysiological mechanism may be secondary neurodegenerative impairment of the cerebral GM mediated by damage to the WM tract; however, further investigation is needed to clarify the underlying processes.

## Data availability statement

The original contributions presented in the study are included in the article/Supplementary material, further inquiries can be directed to the corresponding author.

## Ethics statement

The studies involving human participants were reviewed and approved by Showa University School of Medicine. The patients/participants provided their written informed consent to participate in this study.

## Author contributions

TK, HM, and KO contributed to the conception and design of this research. TK, MH, and MI contributed to the analysis and interpretation of the data as well as the initial drafting of the work. TK, KO, MH, MA, YM, AF, SY, MK, SH, YB, MI, and HM contributed to the acquisition of the data, critically revised it for important intellectual content, and approved its final version and are accountable for the contents of this research. All authors contributed to the article and approved the submitted version.

## Funding

This study was supported by Grants-in-Aid for Scientific Research (Kakenhi) from the Japan Society for the Promotion of Science (JSPS) under Grants 20 K19111 (AF) and JP19K07965 (KO).

## Conflict of interest

The authors declare that the research was conducted in the absence of any commercial or financial relationships that could be construed as a potential conflict of interest.

## Publisher’s note

All claims expressed in this article are solely those of the authors and do not necessarily represent those of their affiliated organizations, or those of the publisher, the editors and the reviewers. Any product that may be evaluated in this article, or claim that may be made by its manufacturer, is not guaranteed or endorsed by the publisher.

## Abbreviations

PvWML: Periventricular white matter lesions
DWML: Deep white matter lesions
ESCI: Early-stage cognitive impairment
BAAD: Brain anatomical analysis using diffeomorphic deformation
GM-V: Gray matter volume
WM-V: White matter volume
LV-V: Lateral ventricular volume
CSF-V: Cerebral spinal fluid volume
TWML-V: Total white matter volume
PvWML-V: Periventricular white matter volume
DWML-V: Deep white matter volume
fWML-V: Frontal lobe white matter lesion volume
3D-SSP: 3D stereotactic surface projection
ACG: Anterior cingulate gyrus
